# High Incidence of Thrombotic Thrombocytopenic Purpura Exacerbation Rate Among Patients With Morbid Obesity and Drug Abuse

**DOI:** 10.7759/cureus.14656

**Published:** 2021-04-24

**Authors:** Preethi Ramachandran, Burak Erdinc, Hesham Ali Abowali, Umar Zahid, Vladimir Gotlieb, Samuel Spitalewitz

**Affiliations:** 1 Hematology and Oncology, Brookdale University Hospital Medical Center, Brooklyn, USA; 2 Internal Medicine, Brookdale University Hospital Medical Center, Brooklyn, USA; 3 Nephrology, Brookdale University Hospital Medical Center, Brooklyn, USA

**Keywords:** thrombotic thrombocytopenic purpura, african american, obesity, drug abuse

## Abstract

This study aims to identify the baseline patient characteristics, clinical presentation, and response to treatment of 11 patients who were diagnosed with thrombotic thrombocytopenic purpura (TTP) between 2014 and 2020 at Brookdale University Hospital Medical Center, Brooklyn, NY. Laboratory and clinical parameters were recorded for 29 patients who received plasmapheresis in this time period. Of 29 patients, 11 had confirmed TTP and one was diagnosed with hereditary TTP. Young, black, and female patients made up the majority of our patient population. A high prevalence of obesity and drug abuse were seen among our patients. Five out of 11 were obese and four of them were morbidly obese; six out of 11 patients were positive for the drug screen including cannabinoids (3), opiates (2), benzodiazepines (1), PCP (1), and methadone (1). Four patients with a positive drug screen had acute kidney injury (AKI), and plasmapheresis helped them enhance their kidney function. We observed a high incidence of AKI and high TTP exacerbation rates in patients who were drug abusers and those who were morbidly obese. There is a paucity of data on the relationship of TTP with obesityor drug abuse and this needs further study.

## Introduction

Thrombotic thrombocytopenic purpura (TTP) is a rare disease with high mortality affecting two people per million per year⁠. It is characterized by thrombotic microangiopathy secondary to thrombocyte aggregation caused by a disintegrin and metalloprotease with thrombospondin type 1 repeats, member 13 (ADAMTS13) deficiency [1,2]⁠. ADAMTS13 deficiency can be acquired or congenital. Acquired TTP is primarily caused by autoantibodies leading to the accumulation of large von Willebrand factor (vWF) multimers causing platelet aggregation and microangiopathic hemolytic anemia (MAHA). TTP classically presents as a pentad: thrombocytopenia, MAHA, kidney injury, neurological involvement, and fever. Even though the “classic pentad” is described to help with the diagnosis of the disease, it is neither sensitive nor specific as in our study. Therefore, the lack of a full pentad should not be used to exclude the diagnosis. Replenishing ADAMTS 13 and removing the associated antibody by plasma exchange is now the mainstay treatment for TTP patients which can achieve remission in approximately 80% of the cases [3]⁠.

## Materials and methods

This study was approved by the Research and Clinical Projects Committee (IRB) of Brookdale University Hospital and Medical Center (BUHMC) on April 28, 2020. A chart review was performed on 29 patients who received plasmapheresis at Brookdale University Hospital and Medical Center in Brooklyn, New York, between 2014 and 2020.

Subjects

Our study included 11 patients with the correct diagnosis of TTP. Diagnostic criteria included (1) clinical manifestations characterized by thrombocytopenia, microangiopathic hemolytic anemia (MAHA) and/or neurological manifestations, fever, acute kidney injury; (2) laboratory investigations included hemolytic anemia, thrombocytopenia, elevated lactate dehydrogenase level (LDH), schistocytes on peripheral smear, negative Coomb’s test, normal prothrombin time (PT) and partial thromboplastin time (PTT); (3) significantly decreased ADAMTS13 activity (<10%); and (4) exclusion of hemolytic uremic syndrome (HUS), disseminated intravascular coagulation (DIC) and other causes of thrombotic microangiopathies (TMA), such as HELLP syndrome, Evans syndrome, and eclampsia.

Clinical data

Clinical data of our cases were collected from HYPERSPACE® Epic 2019 Aug, electronic medical record system by reviewing their demographics, medical history, home medications, presenting symptoms, comorbidities, laboratory parameters, ADAMTS13 level, and treatments received. Clinical features included thrombocytopenia with microangiopathic hemolytic anemia, the presence of acute kidney injury, fever, and neurological symptoms. Laboratory parameters included initial hemoglobin, lowest hemoglobin, reticulocyte count, initial platelet count, lowest platelet count, lactate, lactate dehydrogenase (LDH), serum creatinine on admission, baseline creatinine, alanine aminotransferase (ALT), aspartate transaminase (AST), initial and peak troponin, presence of antinuclear antibody (ANA), ADAMTS13 level and ADAMTS13 inhibitor level.

Measurements of ADAMTS13 activity and antibodies to ADAMTS13

Blood samples to measure ADAMTS13 activity and inhibitor level were collected from the patients before the plasma exchange. ADAMTS13 activity was measured by Quest Diagnostics Nichols Institute, Chantilly, VA, using a chromogenic, enzyme-linked immunosorbent assay (ELISA). Inhibitor testing was performed when ADAMTS13 activity levels are equal to or less than 30%. The patient’s sample is heat-inactivated and mixed with an equal volume of pooled normal plasma (PNP) before testing. After testing, the residual activity is calculated, and the inhibitor concentration is expressed in Bethesda equivalent units (BEU).

Assessment of therapeutic effects

Complete remission was defined as full clinical recovery and recovery of a normal platelet count (>150 x 10^9^/L) for at least two days. Exacerbation was defined as a recurrent disease within 30 days after reaching a treatment response. Relapse was defined as recurrent disease 30 days or longer after reaching a treatment response.

Statistical analysis

Mortality, exacerbation, and relapse rate were described using descriptive statistics. We used median and interquartile range (IQR) as data were not normally distributed. Categorical variables were presented as frequency and percentages. The mean of continuous variables was compared using the Mann-Whitney U test, while for categorical variables Fischer’s exact test was performed as of small data size.

## Results

Eleven patients were diagnosed with TTP (thrombotic thrombocytopenic purpura) at Brookdale University Hospital and Medical Center (BUHMC) between 2014 and 2020. One of the 11 patients had a hereditary form of TTP whereas the rest had acquired TTP. Nine of the 10 patients with TTP were newly diagnosed, and one had relapsed TTP. The median age was 44 years and there was a slight female predominance (64%). The majority of our patients were African Americans (72%) as reflective of our general patient population at BUHMC. Five of 11 patients were obese (BMI >30 kg/m^2^) and four of them were morbidly obese (BMI >40 kg/m^2^). Three patients had autoimmune disorders at the time of diagnosis (two patients with Hashimoto’s thyroiditis and one with antiphospholipid syndrome). Six patients had positive drug screens which included cannabinoids (3), opiates (2), benzodiazepines (1), phencyclidine (PCP, 1), and methadone (1). Only one patient had a history of cancer (cervical cancer in remission). Five of the 11 patients had a history of prior surgeries. The main characteristics of the patients are summarized in Table 1. The presenting symptoms on admission were gastrointestinal (63%) neurological (54%), cardiopulmonary (18%) which all are summarized in Table 2.

**Table 1:** Main characteristics of the patients SD: standard deviation, PCP: phencyclidine

Main characteristics of the patients		
Age, median (range)	44 yrs (22-79 yrs)	
Gender	n	%
Male	4	36.36
Female	7	63.63
Body mass index (BMI), mean ±SD (range)	35.72 ±13.67 (21.7-59.10)	
Ethnicity	n	%
African American	8	72.72
Hispanic	2	18.18
Asian	1	9.09
History of cancer	1	9.09
History of autoimmune disease	3	27.27
Hashimoto thyroiditis	2	18.18
Anti-phospholipid syndrome	1	9.09
Drug abuse	6	54.54
Cannabinoids	3	27.27
Opiates	2	18.18
Benzodiazepines	1	9.09
PCP	1	9.09
Methadone	1	9.09

**Table 2:** Initial presentation TTP: thrombotic thrombocytopenic purpura

	n	%
TTP pentad	1	9.09
Microangiopathic hemolytic anemia	11	100
Thrombocytopenia	11	100
Renal involvement	7	63.63
Neurological involvement	6	54.54
Subdural hematoma	1	9.09
Acute ischemic stroke	1	9.09
Fever	1	9.09
Neurological symptoms	6	54.54
Dizziness	4	36.36
Altered mental status	4	36.36
Headache	3	27.27
Syncope	2	18.18
Seizures	1	9.09
Focal neurologic deficits	1	9.09
Cardiopulmonary symptoms	2	18.18
Chest pain	2	18.18
Shortness of breath	1	9.09
Gastrointestinal symptoms	7	63.63
Nausea or vomiting	5	45.45
Abdominal pain	4	36.36
Loss of appetite	2	18.18
Difficulty swallowing	1	9.09
Diarrhea	1	9.09
Hematemesis	1	9.09
Other symptoms	2	18.18
Easy bruising	1	9.09
Hematuria	1	9.09
Dysuria	1	9.09

Classical pentad of TTP was present in only 9% of our patients. A combination of microangiopathic hemolytic anemia and thrombocytopenia was observed in 100% of the patients. A total of 63% of patients had acute kidney injury, 54.5% had neurological symptoms, and only 9% had fever on admission. Subdural hematoma and acute ischemic stroke were diagnosed in two of the patients who presented with neurological symptoms (Table 2). AKI was observed in four out of six patients with a positive drug screen and one of those was morbidly obese. All patients with drug abuse and AKI had a complete recovery of their renal function after TTP treatment whereas one patient had only partial improvement due to chronic kidney disease at baseline.

All patients had low ADAMTS13 activity of <10%. Ten of the 11 patients had high ADAMTS13 inhibitor levels and one had an undetectable level of inhibitor as the patient had hereditary TTP. The average PLASMIC score⁠ was calculated as six [4]. Mean admission hemoglobin was 9.17 g/dl (standard deviation {SD} 2.57) and the lowest hemoglobin was 6.46 g/dl (SD 1.3). Mean values of other laboratory parameters included reticulocyte count of 0.148 x10^6^/uL (SD 0.10), initial platelet count of 20.72 x10^3^/uL (SD 13.34), lowest platelet count of 10.45 x10^3^/uL (SD 6.05), lactic acid of 3.44 mmol/L (SD 2.43), lactate dehydrogenase (LDH) of 4378 IU/L (SD 2678), creatinine of 1.63 mg/dl (SD 1.03), alanine transaminase (ALT) of 42.18 U/L (SD 40.6), aspartate transaminase (AST) of 85.76 U/L (SD 51.48), initial troponin level of 0.282 mg/ml (SD 0.48), and peak troponin of 2.43 ng/ml (SD 2.91). Laboratory results are summarized in Table 3. 

**Table 3:** Laboratory results SD: standard deviation, LDH: lactate dehydrogenase, ADAMTS13: a disintegrin and metalloproteinase with a thrombospondin type 1 motif, member 13, BEU: Bethesda equivalent units

	Mean ±SD (range)	Reference range
Initial hemoglobin	9.17 ±2.57 (5.7-14.8)	12.9-16.7 g/dl
Lowest hemoglobin	6.46 ±1.30 (5.3-8.8)	
Reticulocyte count	0.148 ±0.10 (0.017-0.351)	0.022-0.090 10^6^/ul
Initial platelet count	20.72 ±13.34 (9-52)	153-328 10^3^/ul
Lowest platelet count	10.45 ±6.05 (6-28)	
Lactate	3.44 ±2.43 (1.2-10.3)	0.70-2.10 mmol/l
LDH	4378 ±2678 (9700-1440)	313-618 IU/l
Creatinine	1.63 ±1.03 (0.95-4.7)	0.66-1.25 mg/dl
Alanine aminotransferase (ALT)	42.18 ±40.60 (11-155)	21-72 U/l
Aspartate aminotransferase (AST)	85.76 ±51.48 (28-194)	17-59 U/l
Initial troponin I	0.282 ±0.48 (0.017-1.570)	0.000-0.034 ng/ml
Peak troponin I	2.43 ±2.91 (0.015-8.140)	
ADAMTS13 activity		
<3%	10	68-163% activity
4%	1	
14%	1	
Inhibitor level	4.94 ±4.69 (0.7-6.7)	<0.4 BEU

All patients with an acquired form of TTP were treated with steroids and plasma exchange. In addition, three of them received immunosuppressive treatment with cyclophosphamide (2) and vincristine (1). One patient with the hereditary form of TTP received treatment with plasma infusions rather than plasma exchange. Three patients had a complicated course with systemic infections: cytomegalovirus bacteremia (1), Proteus mirabilis bacteremia (1), and group B streptococci bacteremia (1). On average, patients received 15 sessions of plasma exchange. Seventy percent of acquired TTP patients received rituximab treatment (4.5 doses on average). Complete remission was observed in 80% of our patients (days to remission: 10.5 days, SD 3.93). But unfortunately, an exacerbation was seen in 70% of the acquired TTP patients. Exacerbation rates were higher in patients with drug abuse as well as in those who were morbidly obese (Table 4). One patient was resistant to plasmapheresis and needed 60 sessions of plasmapheresis in a total of 96 days. Only one out of nine newly diagnosed acquired TTP had relapsed disease (time to relapse: three years). The length of stay was 15 days on average. All patients were discharged home after the initial diagnosis. Three patients died in three, 19, and 23 months after the initial diagnosis.

**Table 4:** Comparison of exacerbation and relapse rates between subgroups TTP: thrombotic thrombocytopenic purpura

	All patients with acquired TTP	Patients with renal involvement	Patients with morbid obesity (BMI>40 kg/m^2^)	Patients with a history of drug abuse
Total, n	10	7	4	5
Exacerbation, %	70.0	57	75	80.0
Relapse, %	20.0	14	50.0	40.0

## Discussion

TTP can be divided into two categories (1) more commonly seen acquired or autoimmune form (94.5% of cases⁠) and (2) very rare hereditary form (4.5% of cases⁠) caused by ADAMTS13 bi-allelic gene mutations also known as Upshaw-Schulman syndrome [5,6]. Severely decreased activity of ADAMTS13, which is primarily synthesized by stellate cells in the liver, is the cause of TTP [7,8]⁠. ADAMTS13 activity levels less than 10% along with clinical features of the disease and laboratory findings are used to make a definitive diagnosis. The main reason for ADAMTS13 deficiency is acquired autoantibodies causing TTP but there may be additional causes that lower the ADAMTS13 levels, such as sepsis, cardiac surgery, pancreatitis, and liver disease [9-13]. An inhibitory antibody is detected in the majority of the cases [7,14]⁠.

TTP typically affects adult populations in their fourth decade of life, and is reported to be more common in young females, African Americans, and patients with a history of autoimmune disease [15]⁠. Our patient population had 63% females, 72% African Americans, and 27% with previously diagnosed autoimmune conditions. The median age in our patient population was 44 years. African American population was the highest among our patient population (73%) compared to Oklahoma (36%) and Harvard registry (20%), respectively [2,16]. This can be explained by the fact that most of our patients in the community we serve are African Americans. Although active cancer and cancer-related chemotherapy have been associated with TTP, one of our patients had a history of cervical cancer that was in remission. Comparison of Harvard and Oklahoma registries with our study is summarized in Table 5.

**Table 5:** Comparison of Harvard and Oklahoma registries with our study (Brookdale registry) TTP: thrombotic thrombocytopenic purpura, ADAMTS13: a disintegrin and metalloproteinase with a thrombospondin type 1 motif, member 13, PEX: plasma exchange

	Brookdale registry	Oklahoma registry [2]	Harvard registry [16]
Geographic region	New York	Oklahoma	Massachusetts
Number of patients	11	78	68
Study population	Clinically suspected cases of TTP with severe ADAMTS13 deficiency	Patients with the first episode of clinically suspected TTP were referred for PEX.	Clinically suspected cases of TTP with severe ADAMTS13 deficiency
Threshold for severe ADAMTS13 deficiency	< 10%	< 10%	< 10%
Female, %	63.63	77	73.5
Median age at initial presentation	44	41	40
Ethnic groups	Black, 72.72%; Hispanic, 18.18%; Asian, 9.09%	White, 62%; Black, 36%; Native American, 2%	White, 63.6%; Black, 19.7%; Asian, 1.5% Hispanic, 15.2%
Clinical findings			
Neurological involvement, %	54.54	67	39.7
Renal involvement, %	63.63	52.5	-
Fever, %	9.09%	10.0	35.3
Laboratory findings			
Hematocrit, %	27.51 (17.1-44.4)	22 (13-28)	27 (23-31)
Platelet count, 9 × 10^9^/L	20.72 (9-52)	11 (5-63)	17 (12-23)
Serum creatinine, mmol/L	1.63 (0.95-4.7)	1.35 (0.8-5.5)	1.08 (0.78-1.47)
Lactate dehydrogenase, IU/L	4378 (9700-1440)	1500 (274-3909)	1107 (797-1349)
Inhibitor positive, %	90.0	88	56
Exacerbation, %	70.0	55	-
Relapse, %	20.0	44	-

TTP causes widespread thrombosis affecting different organ systems, and some are more commonly affected than others [17]. GI symptoms tend to be commonly seen in patients with TTP ranging from 35% to 40% [18]⁠. The range of the GI symptoms in our population was very broad from more commonly seen abdominal pain, nausea, vomiting, and diarrhea to less commonly seen symptoms like dysphagia or hematemesis. GI symptoms in our study population were observed in 64% of the patients which is slightly higher than previously reported studies. The nervous system has been reported to be the most commonly affected visceral organ at presentation, occurring in 67% of cases [2]. The symptoms might be minor such as headache or transient altered mental status (26%) to severe such as focal neurological deficits, seizures, or coma (41%) [2]⁠. In our study, 54% of the patients presented with neurological symptoms ranging from mild to severe (Table 2). Moreover, two patients who presented with severe neurological symptoms such as syncope, focal neurological deficits, and seizures were diagnosed with a subdural hematoma and acute ischemic stroke. The incidence of neurological manifestations was noted to be 39.7% in the Harvard registry and 67% in the Oklahoma registry [2,16]. Renal dysfunction is frequently seen in patients with TTP, more commonly reported in older patients [2,19]⁠. Acute kidney injury was seen in 63% of our patients, which is slightly higher than patients in the Oklahoma registry (52.5%) [2]⁠. Our patient population had a higher incidence of AKI than neurological symptoms compared with other registries which had a higher incidence of neurological manifestations rather than AKI. A detailed analysis into the subgroup of patients with AKI revealed that 57% of them had a positive drug screen and 28% of them had morbid obesity. We observed that the renal functions in most drug abusers (five of six) completely reversed back to normal within one to two weeks after treatment of TTP. Even though baseline renal functions were not available for many of these patients, the observation of improvement of renal function with TTP treatment proves the fact that substance abuse did not play a confounding role in renal impairment. Fever was noted to be common in the Harvard registry, including 35% of their patients [16]⁠. Despite fever being one of the criteria in the classic pentad of TTP, it was rarely seen in our patient population (one of 11 patients). Even though cardiopulmonary symptoms were relatively uncommon, we had two patients (18%) who presented with chest pain and shortness of breath. In addition, we found significantly elevated mean troponin I of 0.282 ng/ml on admission and mean peak troponin I of 2.43 ng/ml (Table 3). Elevated troponin level is associated with a worse prognosis, as reported by Alwan F. et al⁠ in 68% of their patients [20].

The mean hemoglobin, hematocrit, and platelet count in our study group was similar to those reported in other registries except for mean creatinine and LDH levels which were higher in our study population as illustrated in Table 5. Complete remission of TTP was observed in 80% of our patients suggesting a good response to initial therapy (days to remission: 10.5, SD 3.93). However, we found a very high rate of exacerbations (70%) compared to previous TTP studies including the Oklahoma registry which reported an exacerbation rate of 55% [2,21-26]⁠. The relapse rate is 20% in our patient population compared with 44% in the Oklahoma registry [2]⁠. This could be secondary to our limited number of patients who presented to our center.

Our study group had 36% of morbidly obese (BMI >40 kg/m^2^) patients who had significantly lower initial platelet count, high AST, and high initial troponin levels compared to the non-morbidly obese patients (Table 6). Our patients with obesity had high exacerbation (75%) and relapse rates (50%). There is a paucity of data on the relationship of TTP with obesity [27,28]⁠. Therefore, this association needs to be further evaluated. 

**Table 6:** Comparison of laboratory data between morbidly obese and non-morbidly obese LDH: lactate dehydrogenase, ALT: alanine aminotransferase; AST: aspartate aminotransferase Bold values are statistically significant (p-value < 0.05).

Variable	Reference range	BMI<40 (n=7)	BMI≥40 (n=4)	p-Value
Age, median (IQR)		44 (33-75)	41 (35-48)	0.71
Gender, n (%)				
Male		2 (28.57)	2 (50.0)	0.57
Female		5 (71.43)	2 (50.0)	
Initial hemoglobin, median (IQR)	12.9-16.7 g/dl	8.7 (6.5-11.2)	9.1 (8.1-10.65)	0.71
Lowest hemoglobin, median (IQR)		6 (5.3-7.7)	6.4 (5.85-7.65)	0.57
Reticulocyte count, median (IQR)	0.022-0.090 10^6^/ul	0.073 (0.052-0.26)	0.137 (0.108-0.212)	0.45
Initial platelet count, median (IQR)	153-328 10^3^/ul	22 (14-36)	11.5 (9.5-15)	0.07
Lowest platelet count, median (IQR)		9 (7-12)	9.5 (8-10)	0.77
LDH, median (IQR)	313-618 IU/l	2390 (1650-7270)	4495 (4300-5965)	0.25
ALT, median (IQR)	21-72 U/l	26 (23-29)	34.5 (28-96.5)	0.13
AST, median (IQR)	17-59 U/l	40 (36-101)	93 (77-148)	0.08
Initial troponin I, median (IQR)	0.000-0.034 ng/ml	0.021 (0.017-0.121)	0.56 (0.057-1.57)	0.086
Peak troponin I, median (IQR)		0.064 (0.017-5.42)	1.57 (0.169-2.08)	0.73

The other subgroup of patients who had a high exacerbation rate were patients with a positive drug screen. Four of the five patients (80%) with a history of drug abuse had TTP exacerbations in addition to a high incidence of AKI (66%), as previously mentioned (Table 7). It is possible but difficult to prove that these drugs induce the autoimmune reaction of TTP or the causative for increased exacerbation in these patients. Further studies are needed to clarify the relation between drug use and TTP presentation and exacerbation. This may be faced with difficulty due to the rarity of the disease.

**Table 7:** Creatinine levels in patients with positive drug screen *Creatinine reference range: 0.66-1.25 mg/dl

	Baseline creatinine level (mg/dL)*	Creatinine level on admission (mg/dL)*	Creatinine level at the time of remission (mg/dL)*
Patient #1	0.9	0.95	1
Patient #2	N/A	1	0.7
Patient #2	N/A	1.28	1.09
Patient #4	N/A	1.5	0.8
Patient #5	N/A	1.88	1.1
Patient #6	N/A	4.7	3.3

TTP is one of the few hematological emergencies that need to be addressed immediately with accurate diagnosis and initiation of therapy. Therapeutic plasma exchange (TPE), glucocorticoids, and rituximab are the most commonly used efficacious treatments available for TTP. Additional treatments and strategies are being investigated to more effectively treat and prevent exacerbation or relapse. Caplacizumab, a humanized antibody derived from nanoparticles, showed promising results in phase 2 (TITAN) and phase 3 (Hercules) studies and approved by Food and Drug Administration (FDA) on February 2019 for initial treatment of TTP along with plasma exchange and immunosuppressive therapy [29]⁠. The mechanism of action is by inhibiting the interaction between uncleaved vWF multimers and platelets. Additionally, the use of recombinant ADAMTS13 is now offering treatment for people with congenital TTP and offers linear improvement in autoimmune TTP⁠. Interestingly, N-acetylcysteine was found to disrupt the disulfide bonds in the vWF [30]⁠. These new mechanisms of treatment offer great hope to patients diagnosed with TTP, yet further studies are needed to ascertain their efficacy and safety.

## Conclusions

We observed a high incidence of acute kidney injury and high TTP exacerbation rates among patients with morbid obesity and a history of substance abuse. TTP is a rare disease and there is a paucity of data on the relationship of TTP with obesity and drug abuse. This relationship should be further investigated by doing retrospective and prospective studies with larger patient populations. In addition, patients diagnosed with TTP should be counseled for weight loss in case they have obesity (BMI> 30 kg/m^2^) and also be screened and counseled for substance abuse. These interventions might decrease the chance of having TTP exacerbations.
